# Massive Ascites as an Initial Presentation of Systemic Lupus Erythematosus in the Absence of Significant Proteinuria: A Case Report

**DOI:** 10.7759/cureus.90750

**Published:** 2025-08-22

**Authors:** Victoria S Martinez, Keysha N Gonzalez-Ramos, Grissel Rios

**Affiliations:** 1 Rheumatology, University of Puerto Rico, Medical Sciences Campus, San Juan, PRI

**Keywords:** lupus serositis, perihepatic ascites, rheumatology & autoimmune diseases, subnephrotic proteinuria, systemic lupus erythematosus

## Abstract

Systemic lupus erythematosus (SLE) is an autoimmune disease that impacts multiple organ systems, including musculoskeletal, mucocutaneous, hematologic, and renal. Clinical manifestations can range from mild symptoms to severe complications. While gastrointestinal involvement in SLE may occur, abdominal serositis is uncommon, particularly as an initial presentation or without significant proteinuria. This symptom is considered rare and is infrequently reported as an early sign of the disease. We present the case of a 26-year-old female patient who was admitted with recurrent ascites and had no previous history of systemic illnesses. Her symptoms included facial swelling, increased abdominal girth, and shortness of breath. A physical examination revealed periorbital edema and abdominal distension. Initial laboratory tests indicated hypochromic microcytic anemia with no significant proteinuria; a 24-hour urine protein test showed 372.4 mg/24 hours. Elevated inflammatory markers and hypocomplementemia suggested SLE, confirmed by further tests, including positive antinuclear antibodies, anti-double-stranded DNA antibodies, and anti-Smith antibodies. Treatment with intravenous methylprednisolone, hydroxychloroquine, and mycophenolate mofetil resulted in symptom improvement without any further complications. The nonspecific nature and variability of early SLE symptoms can delay diagnosis, underscoring the importance of recognizing atypical presentations. This case highlights the rare occurrence of massive ascites with low-grade proteinuria as an initial sign of SLE, reinforcing the need to include it in the differential diagnosis of unexplained ascites.

## Introduction

Systemic lupus erythematosus (SLE) is a complex autoimmune disease characterized by the production of autoantibodies and the involvement of various organ systems [1]. SLE affects approximately 3.4 million individuals worldwide, with a significantly higher prevalence in women of childbearing age [1]. The clinical manifestations of SLE are diverse, ranging from mild symptoms such as fatigue, malar rash, and joint pain to more severe complications, including renal failure, neurological disorders, and cardiovascular involvement [2]. This case aims to raise awareness of atypical presentations of SLE, particularly in patients presenting with ascites and minimal renal involvement.

A research study documenting the lesser-known clinical manifestations of SLE reports that gastrointestinal involvement has a prevalence of 15% to 60% [2]. Serositis refers to inflammation of serous membranes, which can include the pleura (lungs), pericardium (heart), or peritoneum (abdomen), the latter leading to fluid buildup known as ascites. However, abdominal serositis was not specifically addressed in this study [2]. Furthermore, a 10-year research study describing the disease manifestations of 1,000 patients reported that 16% had serositis, but it was documented as pleuritis and/or pericarditis, not in the peritoneum [3]. 

Ascites, the abnormal accumulation of fluid in the abdominal cavity, is an uncommon initial presentation in SLE, typically associated with advanced disease stages or secondary to nephrotic syndrome or congestive heart failure [4]. A recent retrospective case-control study evaluating serositis in SLE patients found that the prevalence of pleuritis, pericarditis, and peritonitis was 82.1%, 78.6%, and 7.1%, respectively [5]. This study also indicates that the average time for patients to develop any serositis was 4 ± 5.3 years after diagnosis, accompanied by symptoms such as arthritis, photosensitivity, oral ulcers, and nephritis [4].

To our knowledge, only a handful of cases of SLE-associated ascites without nephrotic-range proteinuria have been reported. Previous reports have described peritonitis as an initial manifestation of SLE, but massive ascites without other systemic features remains extremely rare [4,5]. Pseudo-pseudo-Meigs syndrome has also been associated with ascites in SLE, though it typically presents with pleural effusions and elevated CA-125, distinguishing it from isolated peritoneal involvement [6]. To our knowledge, there are no well-documented cases of massive ascites as the initial manifestation of SLE in the absence of significant proteinuria. This case report addresses the unusual clinical manifestation of massive ascites without significant proteinuria as the first sign of SLE. The purpose of this report is to highlight this rare presentation and to emphasize the need to include SLE in the differential diagnosis of unexplained ascites, even in patients without renal involvement.

This article was previously presented as a poster abstract at the American Medical Association's (AMA) 2024 Interim Meeting on November 8, 2024. 

## Case presentation

**Figure 1:**
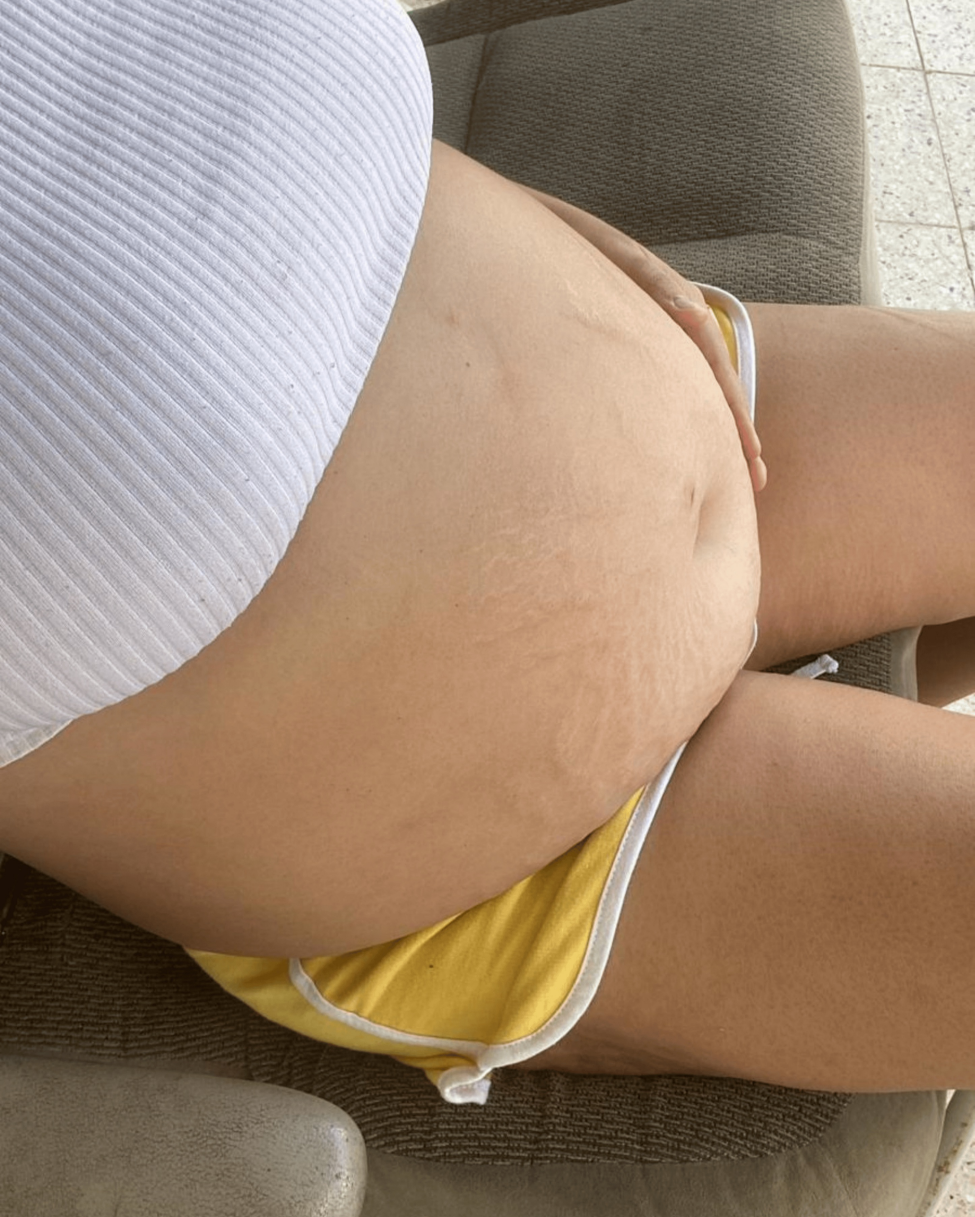
The patient presented with increased abdominal girth on the day of admission.

A 26-year-old woman was admitted to the hospital due to recurrent ascites without any systemic illnesses. Four months before admission, she experienced facial swelling, followed by an increase in abdominal girth and shortness of breath. She had no history of tobacco, alcohol, or recreational drug use. She reported fatigue, weight gain, alopecia, and back pain, denying any other symptoms during the review of systems. Her body mass index was 26.8 kg/m², blood pressure was 87/54 mmHg, temporal temperature was 96.7°F, respiratory rate was 20 breaths per minute, and radial pulse was 99 beats per minute. Physical examination revealed periorbital edema and a distended abdomen (Figure 1). The patient had no oral ulcers or skin rashes and was not in respiratory distress. 

The hematology-oncology team noted an isolated elevation of CA-125 without evidence of malignancy. Echocardiography revealed normal cardiac structure and function, aside from a small pericardial effusion without hemodynamic significance, with no evidence of valvular pathology, intracardiac masses, or functional impairment. The laboratory results showed microcytic anemia without other cytopenias, normal renal and hepatic function, and hypoalbuminemia (Table 1). She had non-nephrotic range proteinuria of 372.4 mg/24 hours. Abdominopelvic computed tomography findings demonstrated signs of anasarca/fluid overload, including ascites and diffuse subcutaneous edema, along with gastric and colonic wall thickening likely due to edema rather than acute gastroenteritis or colitis. The liver shows homogeneous density and a normal outer contour without focal lesions. The patient underwent a large-volume paracentesis with removal of 10 liters of ascitic fluid; cultures were negative for infection.

**Table 1:** Pertinent laboratories and serologies μL: microliter; g: grams; dL: deciliter; fL: femtoliter; pg: picogram; U: units; L: liter; mmol: millimoles; mg: milligrams; IU: international units; mL: milliliter; AI: arbitrary index; APL: anticardiolipin antibody IgA; MPL: anticardiolipin antibody IgM; GPL: anticardiolipin antibody IgG

Test	Result	Normal range	Units
White blood cells	5.30	4.80-10.80	10^3/uL
Hemoglobin	10.9 L	12.0-16.0	g/dL
Hematocrit	34.2	37.0-47.0	%
Mean corpuscular volume	74.8	81.0-99.0	fL
Mean corpuscular hemoglobin	23.9	27.0-31.0	pg
Red cell distribution width	16.9	11.5-14.5	%
Platelet count	256	130-400	10^3/uL
Mean platelet volume	11.3	7.4-10.4	fL
Activated partial thromboplastin time	35.0	23.9-30.7	seconds
Prothrombin time	10.8	9.5-12.1	seconds
International normalized ratio	1.0	0.93-1.15	
Aspartate aminotransferase	13	0-33	U/L
Alanine aminotransferase	9	7-40	U/L
Alkaline phosphatase	57	46-116	U/L
Total protein	3.80	5.70-8.20	g/dL
Albumin	1.5	3.2-4.8	g/dL
Total value Bilirubin	<0.15	0.30-1.20	mg/dL
Lactate dehydrogenase	115	120-246	U/L
Sodium	131	136-145	mmol/L
Calcium	7.10	8.30-10.60	mg/dL
Brain natriuretic peptide	8	0-99	Pg/mL
Urinalysis			
Protein	30	negative	mg/dL
24-hour protein	372.4	<150	mg/24hours
White blood cells	119.9	0.0-23.2	/uL
Bacteria	2127.3	0.0-1933.2	/uL
Serology			
Erythrocyte sedimentation rate (ESR)	99	0-20.0	mm/hr
Rheumatoid factor quantitative	<3.50	0.00-13.99	IU/mL
Complement C3	23.1	84-160	mg/dL
Complement C4	4	12-38	mg/dL
Antinuclear antibody (ANA) direct	Positive	Negative	
Anti-double-stranded DNA (anti-dsDNA) antibody quantitative (QN)	>300	0-9	IU/mL
Ribonucleoprotein (RNP) antibodies	>8.0	0.0-0.9	AI
Smith antibodies	>8.0	0.0-0.9	AI
Sjogren’s anti-SS-A	6.6	0.0-0.9	AI
Thyroid peroxidase	40	0-34	IU/mL
B-2 glycoprotein antibody (Ab) IgA	< 9 APL	< 9 APL	U/mL
B-2 glycoprotein Ab IgM	< 9 MPL	< 9 MPL	U/mL
B-2 glycoprotein Ab IgG	< 9 GPL	< 9 GPL	U/mL
Anticardiolipin Ab IgM	20 MPL	<9 MPL	U/mL
Anticardiolipin Ab IgG	< 9 GPL	<9 GPL	U/mL

The patient was started on intravenous methylprednisolone 1 mg/kg/day (60 mg) due to elevated inflammatory markers, lymphadenopathy, and hypocomplementemia. Further laboratory results, including a lupus panel, confirmed the underlying condition (Table 1). The patient had low complement C4 and a positive antinuclear antibody (ANA) test. Furthermore, her anti-double-stranded DNA (anti-dsDNA) antibodies, ribonucleoprotein (RNP) antibodies, anti-Smith antibodies, Sjögren’s anti-SS-A, and thyroid peroxidase were all elevated. Her Systemic Lupus Erythematosus Disease Activity Index (SLEDAI) score was 12 points at the time of SLE diagnosis.

Without further complications, the patient’s ascites and generalized edema gradually improved with intravenous methylprednisolone, hydroxychloroquine 400 milligrams daily, and mycophenolate mofetil 3 grams daily. Given the constellation of findings and the patient’s systemic involvement, the proteinuria was attributed to early lupus-related renal involvement and not to alternative causes such as heart failure, liver disease, or malignancy. Mycophenolate mofetil was selected due to its favorable efficacy and safety profile for induction therapy in non-severe lupus nephritis and extrarenal disease.

**Figure 2:**
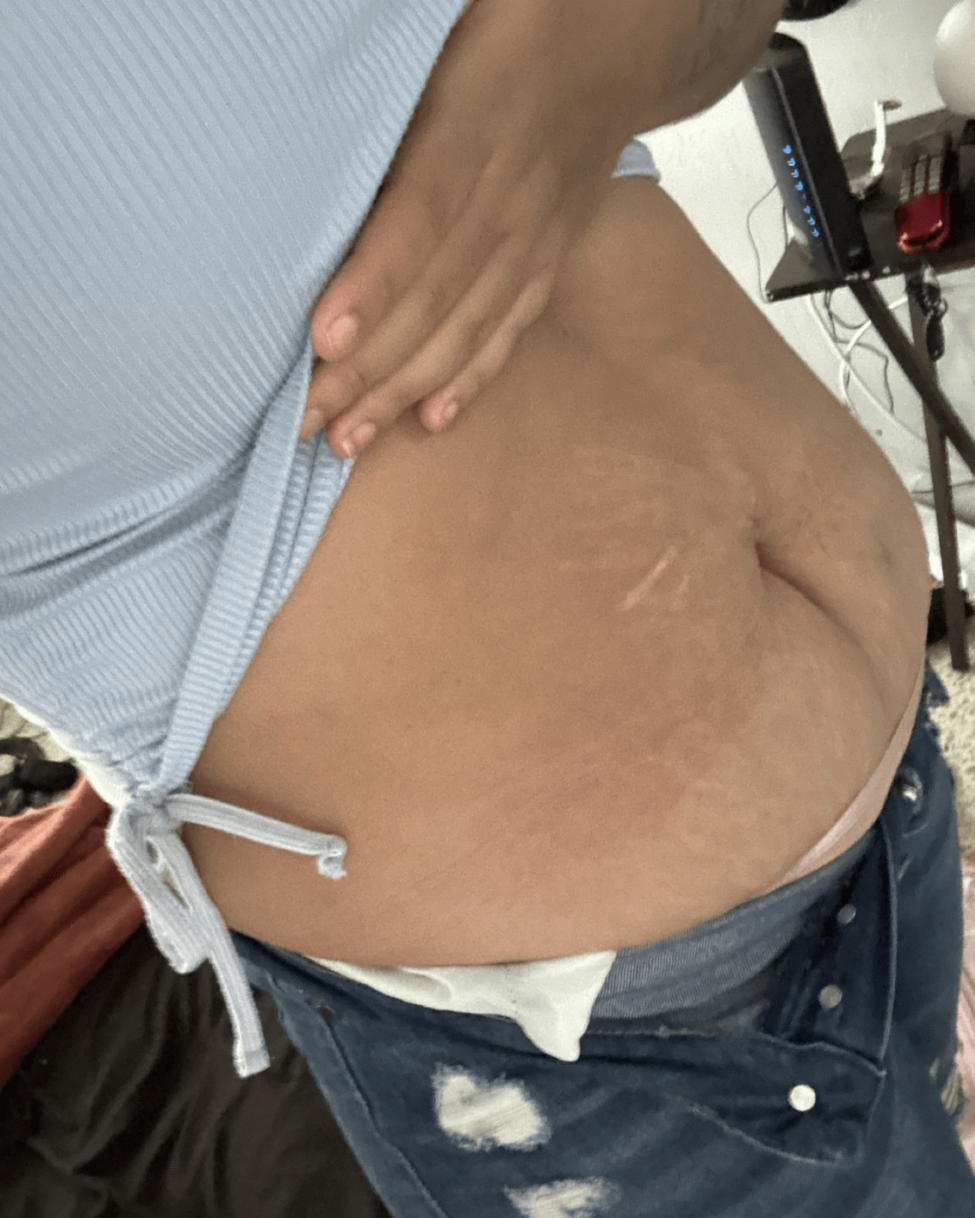
The patient’s decreased abdominal girth at the six-month follow-up after treatment

At the six-month follow-up in the outpatient department, the patient’s ascites had resolved (Figure 2), and she had no systemic symptoms other than photosensitivity and a mild malar rash. Her erythrocyte sedimentation rate (ESR) was 38 mmHg, the protein/creatine ratio was 0.32, anti-dsDNA antibodies were down-trending (84.5 IU/mL), and complement levels were up-trending (C3: 44 mg/dL; C4: 7.4 mg/dL), indicating improvement in disease activity. In addition, her new SLEDAI score was six points. She continued treatment with hydroxychloroquine, mycophenolate mofetil, and prednisone tapering. 

## Discussion

This report documents the unusual clinical manifestation of massive ascites as the presenting symptom of SLE without a significant level of proteinuria. Typical clinical symptoms include fatigue, joint pain, fever, oral ulcers, hair loss, malar rash, photosensitivity, and renal involvement [7, 8]. However, the only symptom our patient experienced was fatigue, which is nonspecific.

The literature has also suggested that ascites related to SLE occurs secondary to renal or cardiac disorders [4,5]. This connection is evident as proteinuria, a hallmark of lupus nephritis, is present in up to 60% of SLE patients and often indicates kidney involvement [7]. Lupus nephritis is classified based on biopsy; however, it is suspected when a patient presents with nephrotic-range proteinuria, defined as more than 500 mg of protein in a 24-hour urine sample, while our patient had 0.3 g. The presence of subnephrotic proteinuria alongside preserved renal function further argued against lupus nephritis as the source of ascites. Instead, low complement levels and elevated anti-dsDNA titers in our patient supported ongoing disease activity and immune complex-driven inflammation. Nonetheless, the accumulation of fluid in our patient's abdomen developed as a primary symptom and was ultimately resolved with SLE treatment.

Reports of SLE presenting with ascites are rare. One case described a 40-year-old Black female patient who developed ascites and joint pain two weeks after surgery, with a prior history of Raynaud’s phenomenon and constitutional symptoms [5]. Examination and laboratory testing confirmed a diagnosis of SLE and pseudo-pseudo Meigs syndrome [5]. In contrast to cases involving pseudo-pseudo Meigs-like features, our patient had no pleural effusions or ovarian masses, and her ascites resolved with immunosuppressive therapy directed at SLE. The resolution of ascites with immunosuppression highlights that immune complex deposition and peritoneal inflammation, rather than secondary organ dysfunction, were the driving mechanisms. Another report involved a 27-year-old male patient who presented with two months of abdominal distension and weakness, accompanied only by weight loss [6]. Rheumatologic evaluation led to a diagnosis of SLE, and the patient responded favorably to corticosteroid treatment [6]. These cases, like ours, highlight ascites as a potential early manifestation of SLE and underscore the role of peritoneal serositis as a contributing mechanism in the absence of significant renal or cardiac involvement.

The underlying pathophysiology of ascites is not fully understood, but it is thought to reflect a form of SLE that predominantly involves the serosal surfaces [4]. Proposed mechanisms include immune complex deposition on the peritoneum, vasculitis of the peritoneal vessels, and lymphoplasmacytic infiltration [4]. These immune-mediated processes can trigger localized inflammation and increase vascular permeability, leading to fluid accumulation in the abdominal cavity. In addition, hypoalbuminemia may have exacerbated ascites formation by lowering oncotic pressure, compounding the effects of serosal inflammation. In cases where renal, hepatic, and cardiac causes are excluded, such serosal inflammation may represent the primary driver of ascites in SLE.

The variability of SLE symptoms presents a diagnostic challenge, particularly when initial manifestations are rare. For instance, in a similar case report, the patient was discharged with a diagnosis of Evans syndrome and treated with prednisone [9]. Despite adhering to treatment, his symptoms continued to worsen [9]. He was readmitted to the hospital and, following additional laboratory tests, was accurately diagnosed and treated for SLE [9]. This illustrates how atypical or nonspecific symptoms in SLE can lead to diagnostic delays, reinforcing the importance of including SLE in the differential diagnosis when presentations are uncommon.

A delay in recognizing ascites as a presenting symptom rather than a condition that occurs only later in the disease progression can worsen the illness. Delayed diagnosis in such atypical presentations may lead to unnecessary testing and prolonged hospital stays.

## Conclusions

This case report aims to highlight the lesser-known clinical manifestations of SLE. We describe a patient with SLE who presented with massive ascites as the first flare or sign of the disease, without significant proteinuria, and demonstrated a good response to immunosuppressive treatment. Clinicians should consider SLE in the differential diagnosis of unexplained ascites, particularly in young women, even in the absence of renal or cutaneous findings.
